# Risk factors associated with Rift Valley fever epidemics in South Africa in 2008–11

**DOI:** 10.1038/srep09492

**Published:** 2015-03-25

**Authors:** Raphaëlle Métras, Chris Jewell, Thibaud Porphyre, Peter N. Thompson, Dirk U. Pfeiffer, Lisa M. Collins, Richard G. White

**Affiliations:** 1 Veterinary Epidemiology, Economics and Public Health Group, Department of Production and Population Health, Royal Veterinary College, Hatfield, United Kingdom; 2 Centre for the Mathematical Modelling of Infectious Diseases and Faculty of Epidemiology and Population Health, London School of Hygiene and Tropical Medicine, London, United Kingdom; 3 Institute of Fundamental Sciences, Massey University, New Zealand; 4 Centre for Immunity, Infection and Evolution, University of Edinburgh, Ashworth Laboratories, Edinburgh, United Kingdom; 5 Epidemiology Section, Department of Production Animal Studies, University of Pretoria, Onderstepoort, South Africa; 6 School of Life Sciences, University of Lincoln, United Kingdom; 7 Tuberculosis Modelling Group, London School of Hygiene and Tropical Medicine, London, United Kingdom

## Abstract

Rift Valley fever (RVF) is a zoonotic and vector-borne disease, mainly present in Africa, which represents a threat to human health, animal health and production. South Africa has experienced three major RVF epidemics (1950–51, 1973–75 and 2008–11). Due to data scarcity, no previous study has quantified risk factors associated with RVF epidemics in animals in South Africa. Using the 2008–11 epidemic datasets, a retrospective longitudinal study was conducted to identify and quantify spatial and temporal environmental factors associated with RVF incidence. Cox regressions with a Besag model to account for the spatial effects were fitted to the data. Coefficients were estimated by Bayesian inference using integrated nested Laplace approximation. An increase in vegetation density was the most important risk factor until 2010. In 2010, increased temperature was the major risk factor. In 2011, after the large 2010 epidemic wave, these associations were reversed, potentially confounded by immunity in animals, probably resulting from earlier infection and vaccination. Both vegetation density and temperature should be considered together in the development of risk management strategies. However, the crucial need for improved access to data on population at risk, animal movements and vaccine use is highlighted to improve model predictions.

Rift Valley fever (RVF) is an emerging zoonotic and vector-borne disease caused by infection with a *Phlebovirus* (Family *Bunyaviridae*). The principal arthropod vectors are mosquitoes from the genera *Aedes* and *Culex*. The major hosts are sheep, cattle and goats, although the disease can also affect camels, buffaloes and many other mammalian species, including humans. The disease is mainly present in Africa, and represents a threat to human health, animal health and production^1^. RVF was first reported in South Africa in 1950, and since then has occurred in the form of irregular outbreaks with long interepidemic periods of up to 15 years^2^.

Between 2008 and 2011, South Africa experienced five RVF outbreak waves: one in 2008, two in 2009, one in 2010 and one in 2011 (Figure 1A). The 2008 and 2009 waves were small (24, 20 and 19 affected farms were reported, respectively) and spatially contained, whereas the 2010 and 2011 waves resulted in 471 and 124 affected farms, respectively, together affecting almost the whole country (Figure 1B–F). In a previous study^3^, three of these five outbreak waves (first 2009 wave, 2010 and 2011) exhibited space-time interaction, which was interpreted as an indication of underlying short and long distance transmission mechanisms. Further research is required to explain the variation in the relative importance of different spatio-temporal transmission processes during those epidemics. Only few studies or field observations have been published on these epidemics and are summarized hereinafter. In 2008, cases were preceded by a period of heavy rainfall^4^, but like other outbreaks in the eastern part of the country, did not develop into a large epidemic. Little published information is available on the 2009 waves, and no unusual environmental conditions were found to be related to the second 2009 wave, although it occurred in an area where flood irrigation was practiced^5^. In 2010 and 2011, RVF epidemics followed periods of heavy rains and localized flooding, and the virus spread almost throughout the whole country.

The aim of the present study was to investigate environmental factors involved in the observed spatial pattern of RVF cases in South Africa across these five outbreak waves. A retrospective longitudinal study was conducted to identify and quantify spatial and temporal environmental factors associated with RVF incidence in South Africa.

## Methods

### Study design and input data

The study area was the whole country of South Africa, and the study period was January 2008 to July 2011. Since RVF is an internationally notifiable disease, RVF data used were outbreak reports collated from the World Animal Health Information Database^6^,^7^,^8^,^9^,^10^, totalling 658 RVF outbreaks in livestock farms (cattle, small ruminants or both). Information on the spatial distribution of livestock farms across the country was not available. Therefore the country was divided into a grid of 45,258 cells, each sized 0.05*0.05 decimal degrees, with these cells being the unit of analysis. Over the study period, time was represented as discrete units of one month each. Since no RVF outbreak was recorded in 2000–07 in South Africa^2^, all grid cells were assumed RVF-negative at the beginning of the study period (January 2008). A grid cell was defined RVF-positive if it had at least one livestock farm (cattle, small ruminants or both) that reported a RVF outbreak during a particular month; resulting in 18 positive cells for the 2008 outbreak, 18 and 17 for the first and second 2009 outbreaks, 436 for 2010 and 121 for 2011. Once positive, a cell was removed (censored) from follow-up.

Potential risk factors were searched by conducting a thorough literature review. The results of this literature review are detailed in Table S1. Factors that were accessible in a geo-referenced format were included in the analysis as covariates (Table 1), and each grid cell was attributed values of those fixed-time and time-varying covariates, the latter reflecting monthly intervals. In total, five time-varying environmental variables, namely Enhanced Vegetation Index (EVI, a measure of vegetation density) of the current and month prior to case (EVI_t_ and EVI_t-1_), monthly average Land Surface Temperature (LST, day temperature) of the current and month prior to case (LST_t_ and LST_t-1_) and vegetation density disturbance (EVI_d_); and two fixed-time topographic variables, i.e. distance from rivers and waterbodies as well as land use, were considered in the analysis.

The dataset was then split into the five outbreak waves in order to conduct a separate analysis for each (January–May 2008, February–June 2009, October-December 2009, January–July 2010 and December 2010 – July 2011). Details on data sources and management are presented in the Supplementary Information.

### Fitting models to data

For each outbreak wave, a spatial semi-parametric Cox model with time-varying covariates using a Besag model for the spatial residual variability was fitted to the data to quantify the effects of potential risk factors on RVF hazard. A spatially correlated model was used to account for the data dependence between neighbouring grid cells. The corresponding hazard function *h_i_*(*t*) for the *i^th^* grid cell, defined up to cell *i*'s infection time *I_i_*, was expressed as follows^11^:


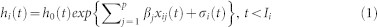
With
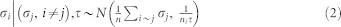
In Equation 1, *x_ij_*(*t*) is the value of the *j^th^* time-dependent variable (or fixed effects) for the *i^th^* grid cell, at time *t*; and *β_j_* is the coefficient of the *j^th^* variable, and *p* the number of variables. The baseline hazard *h*_0_(*t*) is a non-specified positive function of time, which was assumed to be constant over the study period, and represents the hazard function for a grid cell for which all risk factor variables are equal to zero. The Besag model is used to account for the spatial dependence between the grid cells, by adding a spatially structured random effect *σ* = {*σ*_1_, ..., *σ_n_*}. These random effects are defined in Equation 2, with *n_i_* being the number of neighbours of grid cell *i*, *i*~*j* indicating that the two cells *i* and *j* are neighbours^11^, and *τ* being the precision of the random vector.

The fixed effect and the spatial random effect coefficients were estimated from the data by Bayesian inference. The priors assigned to the model coefficients were normally distributed, as were the coefficients of the posterior distributions. In the model presented in Equations 1 and 2, the priors assigned to the fixed parameters were uninformative (normal distribution of precision 0.1 or variance equal to 10), as was the prior distribution assigned to the spatial random effects. For the latter, the precision *τ* was defined as *θ* = log(τ), where the initial values of the hyperparameter *θ* followed a log-gamma distribution (parameter values: scale = 1, shape = 5e−05). Uninformative priors were used so all values could be tested and compared in order to estimate the parameters that fitted the best the data. Using informative priors would have expedited the analyses, but since no previous data were available, choosing arbitrary parameters for priors would have been difficult to justify.

The Bayesian model was fitted using integrated Laplace approximation (INLA) methodology^12^. For large datasets, routine MCMC implementations may be challenging, exhibiting convergence problems and requiring a long computational time. As an alternative, INLA offers fast and accurate results compared to MCMC, as discussed in *Rue 2009*^13^. We note that the use of INLA requires that the latent variable follows a Gaussian distribution, as is the case for the spatial component in our model.

Univariable and multivariable analyses were conducted (see Supplementary Information); models were compared and selected using the deviance information criterion (*DIC*)^14^; the best and most parsimonious model was the one with the smallest *DIC*. The model fit was assessed visually by plotting the observed location of cases on a map of the models' fitted hazard ratio; and by an analysis of the martingale residuals' spatial pattern using semivariograms (Supplementary Information). Statistical analyses were implemented in R version 2.13.1^15^. The R-INLA package was used to fit the Besag spatial models^12^ and the semivariograms were produced from the “geoR” package^16^,^17^.

## Results

Table 2 presents the results of the multivariable analyses for the five outbreak waves. The results of the 2008, first 2009 and 2010 waves exhibited similar patterns, but were different for the second 2009 and the 2011 waves.

In 2008 and the first 2009 wave, increased vegetation density was strongly associated with RVF occurrence (2008: HR (Hazard Ratio) = 111.38, 95% CI (Credibility Interval) [2.38–5411.58]; first 2009 wave: HR = 26.85, 95% CI [9.34–77.18]), as was the presence of wetlands (2008: HR = 6.73, 95% CI [1.71–26.43]; first 2009 wave: HR = 27.57, 95% CI [7.01–108.39]). Similarly, in 2010, RVF hazard also increased with increased vegetation density, regardless of the vegetation density in previous RVF-free years, which means for all categories of EVI disturbance. In addition, in the month of RVF occurrence, only those driest cells which experienced an increase in vegetation density compared with previous RVF-free years appeared to be at increased risk of RVF occurrence (HR = 4.20, 95% CI [1.79–9.84]). Presence of wetlands was also a risk factor for RVF occurrence in 2010 (HR = 3.52, 95% CI [1.34–9.26]). However, for the largest 2010 epidemic, the strongest risk factor was temperature. RVF hazard was increased by 15.71 (95% CI [10.12–24.38]) in areas with temperature between 25°C and 32°C, and by 44.35 (95% CI [28.65–68.67]) in areas with temperature 32°C or above, compared with areas with a temperature below 25°C.

**Figure 1:**
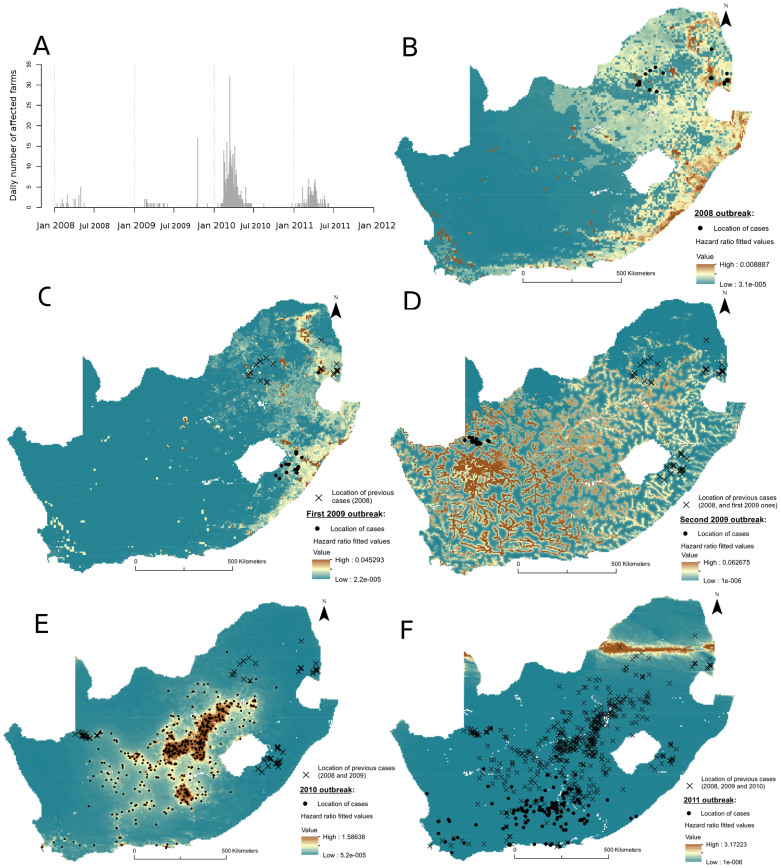
Rift Valley fever 2008–11 epidemics in South Africa. (A) Epidemic curve (daily number of RVF affected farms). Risk maps of the fitted hazard ratio for the (B) 2008, (C) first 2009, (D) second 2009, (E) 2010 and (F) 2011 outbreaks. For each outbreak wave, dots represent the location of affected farms, and crosses the location of previously affected farms. These maps were created using the software ArcGIS version 10.1.

For the second wave of 2009, and in 2011, results exhibited a different pattern. In the second 2009 wave with cases located close to the Namibian border (Figure 1D), RVF hazard was increased with proximity to rivers and waterbodies, but decreased with vegetation density and temperatures. In 2011, RVF hazard was also decreased with vegetation density, but increased with high temperatures.

With regard to the visual analyses of the model fit, the models' fitted values for the 2010 outbreak showed a good fit to the observed data (Figure 1E), but the spatial autocorrelation did not seem to have been removed from the model, especially beyond the distances of about 40 km (0.4 decimal degrees) (Figure S1D). In 2008, the spatial analysis of the martingale residuals exhibited no spatial structure up to 0.4 decimal degrees (Figure S1A); and, although positive cases were located in areas at higher risk, the model showed other high-risk areas in the northern and coastal regions of the KwaZulu-Natal, and the coastal region of the Eastern Cape, where no outbreaks were reported (Figure 1B). In addition, both 2009 models did not show any residual spatial autocorrelation (Figure S1B and S1C), the fitted values of both 2009 models captured the location of reported cases well, but large areas with no reported cases were predicted to be at higher risk (Figure 1C and 1D). In 2011, the residuals did not show evidence of positive spatial autocorrelation (Figure S1E), and the model did not describe the data well, as cases were located in lower risk areas (Figure 1F).

## Discussion

To our knowledge, this is the first study in South Africa which has investigated factors associated with RVF incidence in animals, using epidemic data and in combination with spatial and time-varying environmental conditions.

In 2008, the first 2009 wave, and in 2010, RVF hazard was increased with increased vegetation density and if located near water or wetland areas. Whilst the 2008 and the first 2009 waves reported a small number of cases, during the largest epidemic in 2010, temperature above 25°C appeared to be a major risk factor. In the second wave of 2009, increased temperature and vegetation density were, contrarily, protective; but being further away from rivers or waterbodies decreased RVF hazard. In 2011, RVF hazard also decreased with increasing vegetation density and with higher temperatures. Whilst the factors identified for 2008 and for the first 2009 and 2010 waves were consistent with current knowledge on RVF epidemiology, those identified for the second 2009 wave and the ones in 2011 were not, and potential explanation for this are discussed in the following paragraphs. Finally, with regards to the model fit, although few cases were reported every year except in 2010, the locations of cases were fairly well predicted by the models, except for the 2011 wave; and the 2008 and both 2009 models highlighted some areas at higher risk in which no cases had been reported.

Vegetation density as a proxy for rainfall, together with temperature, were used as indicators of habitat suitability for vectors and viral amplification, and therefore were assumed to indicate a potential for underlying vector-borne transmission of the disease. Beyond the common water-related factors, the difference found between the 2008/first 2009 waves and the one in 2010, was temperature. In 2010, average temperature above 25°C was the most important risk factor. This finding is consistent with experimental RVF virus transmission studies conducted for two RVF vector species (*Culex pipiens* and *Aedes taeniorhynchus*). These studies showed that temperature above 26°C not only shortened the extrinsic incubation period (time interval between ingestion of virus and subsequent transmission by the mosquito) but also increased RVF virus transmission rate^18^; and that these effects were reversed with temperature below 26°C in *Culex pipiens*^19^. As such, temperatures above 26°C may constitute a trigger for RVF epidemics, by amplifying RVF virus transmission in these mosquito species.

In 2010, a set of environmental conditions was observed that may have resulted in improved suitability for vectors' biological cycle and potentially for viral amplification. In 2008 and the first 2009 wave, the number of cases was limited and geographically contained. The absence of sufficient susceptible hosts, the early vaccination implemented on some of the affected and neighbouring farms^6^,^7^, or only short-term presence of suitable environmental conditions may have prevented a widespread epidemic. The 2008 wave, which began close to the Kruger National Park, could have resulted from re-emergence of the virus from reservoir hosts, since the incriminated RVF virus, of lineage C, was similar to the one that caused an outbreak in African buffaloes (*Syncerus caffer*) in the Kruger National Park in 1999^20^. The deposition of vertically infected eggs by mosquitoes that fed on those animals has been suggested as a possible mechanism for virus persistence in this part of the country^21^. The first 2009 wave, in KwaZulu-Natal, was caused also by the same lineage C^20^. It is therefore likely that the virus was imported from the 2008 cluster, and emerged following extensive periods of rain. Indeed, those cases were found to be associated with an increased vegetation density, suggesting the presence of conditions favorable for virus re-emergence, consistent with results from other studies^3^,^4^.

In contrast, for the second 2009 wave, which occurred in the Northern Cape province close to the border with Namibia, vegetation density and increased temperature were found to decrease RVF hazard, and proximity to rivers increased RVF hazard. These findings are consistent with an outbreak investigation that did not observe any specific rainfall events in that area at that time, suggesting that climatic factors were not incriminated, but suggested local flooding related to irrigation techniques as a potential cause of virus emergence^5^. These outbreaks were caused by a RVF virus from lineage H, the same lineage as the one identified in 2004 in Namibia^20^.

In 2011, the results are challenging to interpret, as RVF hazard was increased with increased temperature, but decreased with increased vegetation density. These observed results could have been confounded by the spatial distribution of immune animals, due to prior natural infection (natural RVF infection cause life-long immunity) or vaccination. Indeed, the 2011 cases mainly occurred in the previously least affected areas (Figures 1B–F), likely where most of the susceptible animals remained. In addition, although little data is available on RVF vaccination in South Africa between 2008 and 2011, a very large number of RVF vaccine doses was sold between May 2010 and March 2011^22^. These were used mainly in the first affected areas (Free State and Northern Cape), at the end of the 2010 wave and prior to the 2011 outbreak^22^. Therefore, the fact that the 2011 cases occurred in the lower risk areas (Figure 1E) and that RVF hazard decreased with increasing vegetation density supports the hypothesis that animal immunity (resulting either from natural infection or previous vaccination), and not climatic factors played a major role in the spatial distribution of 2011 cases. To investigate in detail the effect of vaccination, data on temporal and spatial vaccine usage are necessary; unfortunately, this information remains unavailable.

The maps in Figure 1 showed that few cases were reported in areas predicted to be at higher risk (specifically in 2008 and 2009). This observation could have resulted from under-reporting, particularly during the later stages of an epidemic within a known affected region rather than for outbreaks in previously unaffected areas; but the models highlighted areas with environmental conditions potentially suitable for RVF occurrence, if virus was to be introduced. Indeed, these models did not explicitly account for between-farm transmission potential given virus exposure. However, the spatial proximity between grid cells was treated as a form of clustering, therefore artificially accounting for the fact that neighbouring cells were sharing similar attributes resulting from their spatial closeness. These common spatial attributes could be, for example, local transmission of the virus due to vector transmission, and this was well captured by all models which did not exhibit residual positive spatial autocorrelation, except beyond 40 km during the 2010 outbreak (Figure S1D). In that year, the presence of positive spatial autocorrelation beyond 40 km suggests the presence of other spatial processes over further distances, which were not captured by the model, such as wind-carriage of infectious mosquitoes or the movement of infectious animals. Neither movements of infected animals nor of vectors could be accounted for as no data were available.

Another potential explanation for the absence of cases reported from areas that were predicted to be at high risk by the models is the variation in the distribution of susceptible host populations. In the model all grid cells were considered equally susceptible at the beginning of follow-up and hence it assumes that the spatial distribution of farms was homogeneous throughout the country. This presents a necessary over-simplification of reality as no spatial information was available on the location of farms, livestock numbers or type of animal production systems (intensive/extensive, indoors/outdoors). The production system may be as important as the livestock numbers or farm location, specifically for the study of vector-borne diseases where it may have a substantial impact on animal exposure to mosquitoes. In 2007, the South African commercial agriculture census estimated about 40,000 farming units, but this estimate combined farming units with field crops, horticulture and animal production activities^23^, without any further detail. In the absence of such data, the assumption of a homogeneously distributed livestock population is left with few alternatives. However, this highlights the need for targeted data collection to help the development of better-informed models.

Finally, it is acknowledged that the number of cases reported each year, and therefore used in the analyses was rather small, except for the 2010 and 2011 waves. This could have resulted in overfitting, which would necessitate, again a cautious interpretation of the results.

In addition, little is known regarding the spatio-temporal dynamics of vaccine use to control RVF in South Africa; therefore, the impact of vaccination could not be accounted for. Although cells that showed a report of a RVF case were considered “immune” and therefore removed from subsequent analysis, this may have only partly adjusted for the impact of immunization following infection, but not vaccination. Indeed, the implementation of vaccination, in local clusters in 2008 and 2009, and then countrywide in 2010 and 2011^6^,^7^,^24^,^25^, may have resulted in a large number of “immune” cells, which would not have been at risk anymore, thereby confounding the effect estimates in the Cox regression models. To ensure that the results of such a modelling framework may be reproducible over a wider geographic area, data recording the temporal and spatial aspects of vaccination should be published together with disease information.

In conclusion, this study used a novel Bayesian model fitting approach to provide the first quantitative estimates for risk factors of RVF incidence in livestock during epidemics in South Africa. Except for the second wave of 2009 (for which anthropogenic modification of the environment may have been the main contributor of the epidemics), an increase in vegetation density was the most consistent risk factor across outbreak waves, until the largest epidemic in 2010. In addition, increased temperature was an important environmental risk factor, and therefore should be considered as a key parameter, along with vegetation density, in further quantitative analyses on RVF. It is, however, critical that information on population at risk, animal movements and spatial and temporal patterns of vaccine use are considered when attempting forecast RVF epidemics.

## Author Contributions

R.M., C.J., T.P., L.M.C. and R.G.W. designed the analyses. R.M. conducted the analyses, created all figures and wrote the main manuscript text. C.J., T.P., P.N.T., D.U.P., L.M.C. and R.G.W. wrote and reviewed the manuscript.

## Supplementary Material

Supplementary Information Supplementary Information

## Figures and Tables

**Table 1:** Input and type of covariates used in the spatial statistical models, units of measurements and data source

Input covariates	Unit	Type of covariate	Source (all data used are publicly available)
**Environmental factors influencing host exposure to mosquito vectors**			
Environmental conditions of the month current to case			
EVIt	EVI index (0–1)	Time-varying	Terra MOD13C2.005 product (2007–2010) (http://modis.gsfc.nasa.gov/)
LSTt	Degree Celsius		Terra V5 MOD11C3.005 (2007–2010) (http://modis.gsfc.nasa.gov/)
Environmental conditions of the month prior to case			
EVIt-1	EVI index (0–1)	Time-varying	Terra MOD13C2.005 product (2007–2010) (http://modis.gsfc.nasa.gov/)
LSTt-1	Degree Celsius		Terra V5 MOD11C3.005 (2007–2010) (http://modis.gsfc.nasa.gov/)
EVId	n/a		Terra MOD13C2.005 product (2000–2007) (http://modis.gsfc.nasa.gov/)
**Topographic factors influencing host exposure to mosquito vectors**			
Distance to rivers and waterbodies	Decimal degrees	Fixed-time	Rivers in Africa (Derived from Hydrosheds) (2010) http://www.fao.org/geonetwork/srv/en/main.home Harmonized DCW-VMap0 Surface Water Bodies (2006) http://www.fao.org/geonetwork/srv/en/main.home
Land use	Agro-pastoralism, Forestry, Herbaceous/bare areas, Irrigated areas, Urban areas, Water/Wetlands		Land Use Systems of the World – Sub Saharan Africa, Beta Version (2008) http://www.fao.org/geonetwork/srv/en/main.home

EVIt = Enhance Vegetation Index of the month current to case; EVIt-1 = Enhance Vegetation Index of the month prior to case; EVI_d_ = Enhanced Vegetation Index disturbance; LSTt = Land Surface Temperature of the month current to case; LSTt-1 = Land Surface Temperature of the month prior to case.

**Table 2:** Results of the multivariable Cox regression analyses for the five 2008–11 waves. Mean values of the hazard ratios (HR) posterior distribution and their 95% credibility intervals (CI)

2008 wave (18 cases)			
*Variables*	*Unit/category*	*HR*	*95% CI*
EVIt	Index (0–1)	113.38	2.38–5411.58
Land use	Agro-Pastoralist	1	NA
	Forestry	1.04	0.35–3.07
	Herbaceous/Bare	0.25	0.06–0.97
	Urban areas		
	Irrigated areas	6.73	1.71–26.43
	Water/Wetlands		

2009, first wave (18 cases)			
*Variables*	*Unit/category*	*HR*	*95% CI*
EVIt	<0.40	1	NA
	≥0.40	26.85	9.34–77.18
Land use	Agro-Pastoralist	1	NA
	Forestry	1.38	0.35–5.43
	Herbaceous/Bare	0.85	0.17–4.31
	Urban areas	3.46	0.60–19.93
	Irrigated areas	27.57	7.01–108.39
	Water/Wetlands		

2009, second wave (17 cases)			
*Variables*	*Unit/category*	*HR*	*95% CI*
EVIt	Index (0–1)	0.00	0.00–0.01
LSTt-1	<25°C	1	NA
	≥25°C	0.00	0.00–0.02
Distance to rivers or water bodies	km	0.81	0.71–0.91

2010 wave (436 cases)				
*Variables*		*Unit/category*	*HR*	*95% CI*
EVId<1	EVI_t_	0 to <0.10	1	NA
		≥0.10 to <0.20	1.06	0.59–1.89
		≥0.20 to <0.25	3.11	1.55–6.23
		≥0.25	7.92	3.87–16.23
1<EVId<1.1	EVI_t_	0 to <0.10	1.14	0.44–2.94
		≥0.10 to <0.20	1.15	0.25–5.26
		≥0.20 to <0.25	2.98	0.61–14.66
		≥0.25	6.69	1.39–32.18
EVId>1.1	EVI_t_	0 to <0.10	4.20	1.79–9.84
		≥0.10 to <0.20	1.38	0.34–5.59
		≥0.20 to <0.25	1.47	0.33–6.55
		≥0.25	4.90	1.15–20.83
LSTt		<25°C	1	NA
		≥25°C to <32°C	15.71	10.12–24.38
		≥32°C	44.35	28.65–68.67
Land use		Agro-Pastoralist	1	NA
		Forestry	0.55	0.23–1.34
		Herbaceous/Bare	0.56	0.38–0.82
		Urban areas	0.92	0.36–2.33
		Irrigated areas	1.13	0.67–1.90
		Water/Wetlands	3.52	1.34–9.26

2011 wave (121 cases)			
*Variables*	*Unit/category*	*HR*	*95% CI*
EVIt-1	Index (0–1)	0.00	0.00–0.00
LSTt	<25°C	1	NA
	≥25°C to <32°C	2.50	1.36–4.61
	≥32°C	2.09	1.00–4.39

EVId: Enhanced Vegetation Index Disturbance; EVI = Enhanced Vegetation Index; LST: Land Surface Temperature.
