# Supplementary material for: Risk factors associated with Rift Valley fever epidemics in South Africa in 2008–11
Source: Sci Rep. 2015 Mar 25;5:9492. doi: 10.1038/srep09492 (PMC4372659; doi:10.1038/srep09492)
Supplement: Supplementary Information [file srep09492-s1.pdf]

Supplementary Information

Risk factors associated with Rift Valley fever epidemics in South Africa in 2008-11

Raphaëlle Métras[1,2*], Chris Jewell[3], Thibaud Porphyre[4], Peter N. Thompson[5], Dirk U. Pfeiffer[1], Lisa M. Collins[6], Richard G. White[2,7]

[1] Veterinary Epidemiology, Economics and Public Health Group, Department of Production and Population Health, Royal Veterinary College, Hatfield, United Kingdom

[2] Centre for the Mathematical Modelling of Infectious Diseases and Faculty of Epidemiology and Population Health, London School of Hygiene and Tropical Medicine, London, United Kingdom

[3] Institute of Fundamental Sciences, Massey University, New Zealand

[4] Centre for Immunity, Infection and Evolution, University of Edinburgh, Ashworth Laboratories, Edinburgh, United Kingdom

[5] Epidemiology Section, Department of Production Animal Studies, University of Pretoria, Onderstepoort, South Africa

[6] School of Life Sciences, University of Lincoln, United Kingdom

[7] Tuberculosis Modelling Group, London School of Hygiene and Tropical Medicine, London, United Kingdom

* Corresponding author:

E-mail: raphaelle.metras@lshtm.ac.uk

SI - Material and Methods

Input data

A literature review was conducted to identify potential risk factors (Table S1). Potential risk factors that were accessible in a geo-referenced format were included in the analysis (Table 1, main manuscript).

Data source

Spatial data on EVI and LST were sourced from the Moderate Resolution Imaging Spectroradiometer (MODIS) website (http://modis.gsfc.nasa.gov/). For EVI (MODIS band 7) all available monthly raster maps, i.e. between February 2000 and August 2011, were extracted from the product Terra MOD13C2.005, totalling 139 monthly maps for each variable. For LST, data for the period March 2000 to August 2011 were available (136 maps) and downloaded from the product Terra V5 MOD11C3.005. Spatial data on land use, rivers and waterbodies were obtained from the Food and Agriculture Organization (FAO) of the United Nations GeoNetwork webportal (http://www.fao.org/geonetwork/srv/en/main.home). ArcGIS version 10 was used to work with global MODIS maps and extract South African data.

Data management

Rainfall for the current and the month prior to case occurrence were potential risk factors for RVF as they influence habitat suitability for vectors [1-9]. However, the Enhanced Vegetation Index (EVI), a measure of vegetation abundance related to rainfall [10, 11], may reflect more accurately vectors' habitat than rainfall value itself. In addition, rainfall is often extremely localized whereas weather stations are very sparsely distributed in many areas; therefore we considered that EVI would give a better indication of local rainfall. Therefore, the EVI value of the month prior to RVF case occurrence (noted EVI_{t-1}), and the EVI value of the month of RVF case occurrence (noted EVI_t) were input covariates. Similarly, temperature is known to influence *Culicidae* mosquitoes' biology [12, 13], and therefore is likely to be a risk factor for RVF outbreaks. The values of monthly average day temperature (Land Surface Temperature, LST) for the month of RVF case occurrence (LST_t) and the one prior to it (LST_{t-1}) were considered. Finally, an index measuring the disturbance of EVI (EVI_d) was also computed to capture the fact that RVF outbreaks seemed to follow periods of unusually heavy rainfall. Disturbance was defined as deviation in EVI value from EVI values recorded during previous RVF-free outbreak years (2000-07), therefore EVI_d was the ratio of EVI

during the month prior to RVF case occurrence, divided by the average of the monthly EVI values for that same month in previous RVF-free years (during 2000-07). In summary, five environmental variables, for which values were allowed to vary on a monthly basis, were considered in the analysis: EVI_t , EVI_{t-1}, LST_t , LST_{t-1} and EVI_d.

Two topographic variables, namely distance from rivers and waterbodies, and land use, were included as non-time varying variables. Distances to rivers and waterbodies were considered since RVF tends to be reported in areas near lakes, and waterbodies, and in riverine areas, which provide favourable habitat for RVF vectors [1, 2, 4, 6, 14, 15, 16, 17]. The variable "distance from rivers and waterbodies" was computed first by converting the two vector layers "distance from rivers" and "waterbodies" into raster files. Then, for each grid cell of the country, the distance between each grid cell centroid to the nearest "river" or "waterbody" grid cell was considered.

Finally, Land use was included in the model, as a fixed-time variable since no yearly data was available, and categorised into agro-pastoral areas, forestry, herbaceous or bare areas, irrigated areas, urban areas and water/wetlands. Finally, the dataset was split into the five outbreak waves in order to conduct a separate analysis for each (January-May 2008, February-June 2009, October-December 2009, January-July 2010 and December 2010 - July 2011).

Model selection

For each outbreak wave, an univariable and multivariable analyses were conducted. Models were compared and selected using the deviance information criterion (*DIC*) [37], which accounts for both model deviance and number of parameters. The best and most parsimonious model was the one with the smallest *DIC*.

For the univariable analyses, the variables LST_t, LST_{t-1} and EVI_d were categorised. For LST_t and LST_{t-1}, cut-off values were 15°C, 25°C, 32°C, based on experimental temperatures used to study infection and transmission rates of RVF virus in *Culicidae* mosquitoes [12, 13]. For EVI_d, cut-off values were 1 and 1.1, therefore enabling to compare cells with a moderate and important increase of vegetation density, with those experiencing a decrease in their vegetation density (EVI_d < 1). The three variables EVI_t, EVI_{t-1} and distance to waterbodies and rivers, were tested as continuous and categorical, for which cut-off values were chosen according to the first quartile, median and third quartile of the variables' distribution. When one of these quartile intervals exhibited zero cases (especially for the outbreak waves with

few cases), it was merged with the immediate above or below quartile, without modifying the cut-off value itself.

Whether variables would be included in the multivariable analyses as continuous or categorical was also based on the *DIC* value. Correlations between variables were checked using the Spearman rank statistics, and variables with a correlation coefficient above the absolute value of 0.7 were considered as correlated. The choice between the two correlated variables was also based on the *DIC* value.

Finally, the variables selected in the univariable analyses were fitted together, and a multivariable analysis for each outbreak wave was carried out. For each outbreak wave, the best and most parsimonious multivariable model was the one with the smallest *DIC* value.

Model diagnostics

The martingale residuals correspond to the difference between the observed and the predicted values. For each grill cell *i*, the martingale residual r_{Mi} is defined as in *Collett 2003* [18]:

$$r_{Mi} = \delta_i - H_0(t_i) * exp\{\sum_{j=1}^{p} \beta_j x_{ij}\,(t_i) + \sigma_i(t_i)\} \quad \text{(Equation S1)}$$

where $x_{ij}(t_i)$ are the values of the explanatory variables for the ith grid cell at time t_i, β_j the estimated coefficients, p the number of variables, σ_i the value of the random effect at time t_i, δ_i an event indicator that takes the value of one if the grid cell is censored and zero otherwise, $H_0(t_i)$ the estimated cumulative baseline hazard up to time t_i..

The assumption of spatial independence, i.e. that residuals were randomly distributed in space was comparing the empirical semivariogram with a simulation envelope expressing spatial independence [19]. The empirical semivariogram plots the spatial dependence of the residuals (y-axis) at pre-defined separating distances (or spatial lags), on the x-axis. Spatial dependence is expressed by the semi-variance *γ(h):*

$$\gamma(h) = \frac{1}{2\,|N(h)|} \sum_{N(h)} Z(S_i) - Z(S_j) \qquad \text{(Equation S2)}$$

Where $|N(h)|$ is the number of distinct pairs of points separated by distance h; $Z(S_i)$ and $Z(S_j)$ the residual values for points i and j. The semi-variance defined above assumes a stationary and isotropic process (when spatial dependence varies only with distance, but not with

direction) and is then used to generate omnidirectional semivariograms. The maximum separating distance used was 1 decimal degree (111km), since the maximum spatial dependence likely to have resulted from transmission was assumed to be 90km [20].

SI - Results

Spatial analyses of the residuals

In 2008, the spatial analysis of the martingale residuals exhibited no spatial structure up to 0.4 decimal degrees (Figure S1A), which means that for those distances, the model showed a good fit to the data. Both 2009 models did not show any residual spatial autocorrelation (Figures S1B and S1C). For the 2010 outbreak, spatial autocorrelation did not seem to have been removed from the model, especially beyond the distances of about 40 km (0.4 decimal degrees) (Figure S1D). Finally, in 2011, the residuals did not show evidence of positive spatial autocorrelation (Figure 1SE).

SI - References

1. Alexander, R.A.. Rift Valley fever in the Union. *J S Afr Vet Med Assoc* **22**, 105-109 (1951).

2. van der Linde, N.T.. A recent epidemic of Rift Valley fever in the Orange Free State. *J S Afr Vet Med Assoc* **24**, 145-148 (1953).

3. Coetzer, J.A. The pathology of Rift Valley fever. 1. Lesions occurring in natural cases in new-born lambs. *Onderstepoort J Vet Res* **44**, 205-211 (1977).

4. van Velden, D.J., Meyer, J.D., Olivier, J., Gear, J.H. and McIntosh, B.. Rift Valley fever affecting humans in South Africa: a clinicopathological study. *S Afr Med J* **51**, 867-871(1977).

5. McIntos, B.M., Jupp, P.G., dos Santos, I. and Barnard, B.J.. Vector studies on Rift Valley Fever virus in South Africa. *S Afr Med J* **58**, 127-132 (1980).

6. Pienaar, N.J. and Thompson, P.N. The history of Rift Valley fever in South Africa. 9th annual congress of the Southern African Society for Veterinary Epidemiology and Preventive Medicine. Aug 18-20, 2010, Pretoria, South Africa. Pages 6-11 (2010).

7. ProMED-mail. Rift Valley fever - South Africa (03): (FS, EC, NC, GT, MP). ProMED-mail 2010; 21 Mar (2010): 20100321.0902 http://www.promedmail.org (Accessed 15 September 2010).

8. ProMED-mail. Rift Valley fever, sheep - South Africa: (FS) OIE. ProMED-mail 2010; 25 Feb (2010): 20100225.0622 http://www.promedmail.org. (Accessed 13 September 2010).

9. ProMED-mail. Rift Valley fever - South Africa (09): multi-province. ProMED-mail 2010; 07 Apr (2010): 20100407.1119 http://www.promedmail.org. (Accessed 15 September 2010).

10. Huete, A., et al. Overview of the radiometric performance of the MODIS vegetation indices. *Remote Sens Environ* **83**, 195–213 (2002).

11. Jamali, S., Seaquist, J.W., Ardö, J. and Eklundh, L. Investigating temporal relationships between rainfall, soil moisture and MODIS-derived NDVI and EVI for six sites in Africa. 34th International Symposium on Remote Sensing of Environment. The GEOSS Era: Towards Operational Environmental Monitoring. April 10-15, 2011, Sydney, Australia (2011).

12. Turell, M.J., Rossi, C.A. and Bailey, C.L. Effect of extrinsic incubation temperature on the ability of *Aedes taeniorhynchus* and *Culex pipiens* to transmit Rift Valley fever virus. *Am J Trop Med Hyg* **34**, 1211-1218 (1985).

13. Brubaker, J.F. and Turell, M.J. Effect of environmental temperature on the susceptibility of Culex pipiens (Diptera: Culicidae) to Rift Valley fever virus. *J Med Entomol* **35**, 918-921 (1998).

14. Gear, J., De Meillon, B., Measroch, V., Davis, D.H. and Harwin, H. Rift valley fever in South Africa. 2. The occurrence of human cases in the Orange Free State, the North-Western

Cape Province, the Western and Southern Transvaal. B. Field and laboratory investigation. *S Afr Med J* **25**, 908-912 (1951).

15. Joubert, J.D., Ferguson, A.L. and Gear, J. Rift Valley fever in South Africa: 2. The occurrence of human cases in the Orange Free State, the north-western Cape province, the western and southern Transvaal. A Epidemiological and clinical findings. *S Afr Med J* **25**, 890-891 (1951).

16. Gear, J., et al. Rift Valley fever in South Africa. A study of the 1953 outbreak in the Orange Free State, with special reference to the vectors and possible reservoir hosts. *S African Med Jour* **29**, 514-518. (1955).

17. Kokernot, R.H., Smithburn, K.C. and Kluge, E. Neutralizing anitbodies against arthropod-borne viruses in the sera of domestic quadrupeds ranging in Tongaland, Union of South Africa. *Ann Trop Med Parasit* **55**, 73-85 (1961).

18. Collett, D. In *Modelling survival data in medical research 2nd edn* (eds Chapman & Hall/CRC), Ch 4, 111-120 (Boca Raton, Florida, 2003).

19. Pfeiffer, D.U., et al. In *Spatial Analysis in Epidemiology* (eds Oxford University Press), Ch 6, 67-80 (2008).

20. Metras, R., et al. Exploratory space-time analyses of Rift Valley fever in South Africa in 2008-2011. *PLoS Negl Trop Dis* **6**, e1808 (2012).

21. Lancelot, R. et al. [Descriptive epidemiology of Rift Valley fever in small ruminants in Southern Mauritania after the 1988 rainy season]. *Rev Elev Med Vet Pays Trop* **42**(4): 485-491 (1990).

22. Chevalier, V. et al. Exposure of sheep to mosquito bites: possible consequences for the transmission risk of Rift Valley Fever in Senegal. *Med Vet Entomol* **18**(3): 247-255 (2004).

23. Chevalier, V., Thiongane, Y. and Lancelot, R. Endemic transmission of Rift Valley fever in Senegal. *Transbound Emerg Dis* **56**(9-10): 372-374 (2009).

24. Elfadil, A.A., Hasab-Allah, K.A. and Dafa-Allah, O.M. Factors associated with Rift Valley fever in south-west Saudi Arabia. *Rev Sci Tech* **25**, 1137-1145 (2006).

25. Linthicum, K.J., Bailey, C.L., Davies, F.G. and Tucker, C.J. Detection of Rift-Valley Fever Viral Activity in Kenya by Satellite Remote-Sensing Imagery. *Science* **235**, 1656-1659 (1987).

26. Clements, A.C. et al. Spatial risk assessment of Rift Valley fever in Senegal. *Vector Borne Zoonotic Dis* **7**, 203-216 (2007).

27. Linthicum, K.J. et al. Climate and satellite indicators to forecast Rift Valley fever epidemics in Kenya. *Science* **285**, 397-400 (1999).

28. Anyamba, A., Linthicum, K.J., Mahoney, R., Tucker, C.J. and Kelley, P.W. Mapping potential risk of Rift Valley fever outbreaks in African savannas using vegetation index time series data. *Photogramm. Eng. Remote Sens* **68**, 137-145 (2002).

29. Anyamba, A., Linthicum, K.J. and Tucker, C.J. Climate-disease connections: Rift Valley Fever in Kenya. *Cad Saude Publica* **17** Suppl, 133-140 (2001).

30. EFSA Panel on Animal Health and Welfare. Opinion of the Scientific Panel on Animal Health and Welfare (AHAW) on a request from the Commission related to "The Risk of a Rift Valley Fever Incursion and its Persistence within the Community". *The EFSA Journal* 238: 1–128. *DOI:110.2903/j.efsa.2005.2238* (2005).

31. AFSSA, Agence Française de Sécurité Sanitaire des Aliments. Avis de l'Agence française de sécurité sanitaire des aliments sur le risque de propagation de la fièvre de la vallée du Rift (FVR) dans un département et une collectivité départementale français de l'Océan Indien (la Réunion et Mayotte), 156 pp (2008).

32. Sellers, R.F., Pedgley, D.E. and Tucker, M.R. Rift Valley fever, Egypt 1977: disease spread by windborne insect vectors? *Vet Rec* **110**, 73-77 (1982).

33. Davies, F.G. Risk of a rift valley fever epidemic at the haj in Mecca, Saudi Arabia. *Rev Sci Tech* **25**, 137-147(2006).

34. Abdo-Salem, S. et al. Risk assessment of the introduction of Rift Valley fever from the Horn of Africa to Yemen via legal trade of small ruminants. *Trop Anim Health Prod* **43**, 471-480 (2011).

35. McIntosh, B. and Jupp, P.G. Epidemiological aspects of Rift Valley fever in South Africa with references to vectors. *Contr Epidem Biostat* **3**, 92-99 (1981).

36. OIE, World Organisation for Animal Health. Rift Valley fever. Follow-up report No. 15. (2010). OIE Ref: 9559, Report Date: 30/07/2010, Country: South Africa. Available at http://www.oie.int/wahis_2/public/wahid.php/Reviewreport/Review?reportid=9559. (Accessed 11 March 2013).

37. Spiegelhalter, D.J., Best, N.G., Carlin, B.P. and van der Linde, A. Bayesian measures of model complexity and fit (with discussion). *J. R. Statist. Soc. B* **64**, 583–639 (2002).

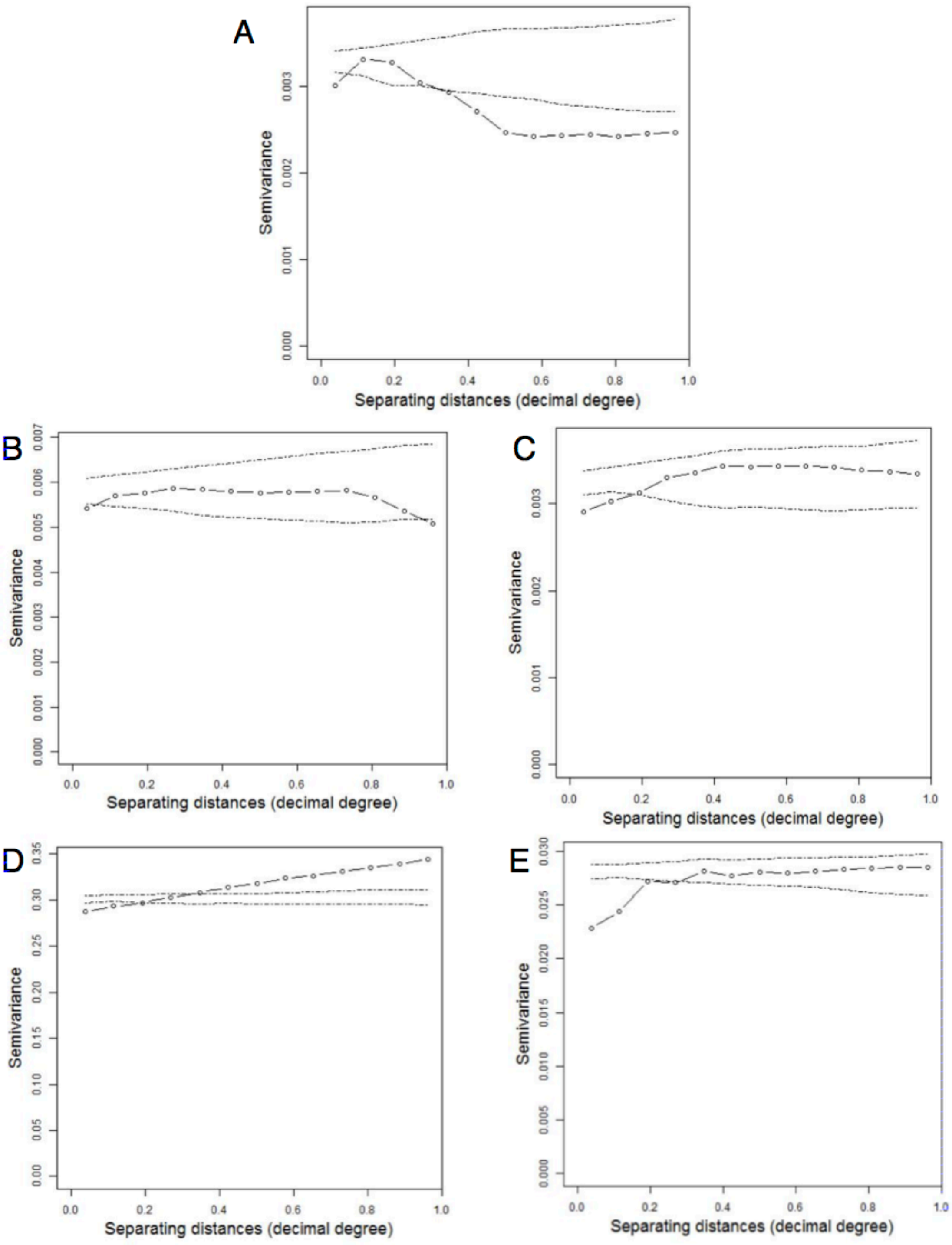

Figure S1: Semivariogram based on the martingale residuals, for the 2008 (A), first 2009 (B), second 2009 (C), 2010 (D) and 2011 (E) outbreaks. The line with empty circles represents the values of the semivariance for pairs of points at increasing separating distances (spatial lags *h*); the two dashed lines represent the 95% simulation envelope of the semivariance. The figures were created using the software R version 2.13.1.

Table S1: List of known risk factors for Rift Valley fever occurrence and pathways for RVF spread; and hypothesized risk factors specific to South-Africa following field observations

Risk factors	Country	Relationship between risk factor and RVF	Reference
**Known risk factors**			
Water-bodies	Senegal	RVF incidence higher in areas with waterbodies	[21]
	Senegal	RVF prevalence & incidence are heterogeneous among ponds	[22, 23]
	Saudi Arabia	RVF prevalence higher with presence of lakes and/or ponds	[24]
Rainfall	Saudi Arabia	RVF prevalence higher with higher rainfall	[24]
	Kenya	RVF mosquito abundance correlated with higher rainfall	[25]
	Senegal	RVF risk higher in areas with higher rainfall	[26]
Mosquito	Saudi Arabia	RVF prevalence higher with higher mosquito density	[24]
NDVI/Vegetation	Kenya	RVF mosquito abundance correlated with higher NDVI	[25]
	Kenya	RVF epizootics associated with increased NDVI	[27]
	Africa	RVF activity associated with 3-months mean NDVI anomalies	[28]
Temperature	Kenya	RVF epidemics associated with positive anomalies SSTs	[27, 29]
**Known risk pathways for RVF virus spread**			
Movements of infected animals	Several countries	Animal movement through trade is potential pathway of virus spread (Egypt, Europe, Saudi Arabia, Mayotte Islands, Yemen)	[30-34]
Mosquito dispersal	Egypt	Insects carried through wind is potential pathway of virus spread	[32]
**Hypothesized risk factors specific to South African context**			
Rainfall	South Africa	Wetter climatic conditions favour vector breeding	[3]
	South Africa	Epidemics in abnormally wet years	[5]
	South Africa	Heavy rainfall creating favourable condition for mosquito breeding	[7]
	South Africa	Heavy rainfall during previous year	[1]
	South Africa	Heavy rainfall	[4, 6]
	South Africa	Heavy rains the previous month	[2, 9]
Water-bodies	South Africa	RVF reported in areas near full lakes and pans	[1, 2, 4, 6, 14, 15]
	South Africa	RVF reported close to large pans	[16]
Rivers & water-bodies	South Africa	RVF present in riverine-pan areas	[17]
Temperature	South Africa	Epidemics area in low temperature area	[35]
	South Africa	Decrease in temperature ended outbreak 2010	[36]
	South Africa	RVF continues over warm winters to next season	[6]

AFSSA = Agence Francaise de Sécurité Sanitaire des Aliments, EFSA = European Food Safety Authority, NDVI = Normalized Difference Vegetation Index, RVF = Rift Valley fever
